# Transcriptome-wide association study of treatment-resistant depression and depression subtypes for drug repurposing

**DOI:** 10.1038/s41386-021-01059-6

**Published:** 2021-06-22

**Authors:** Chiara Fabbri, Oliver Pain, Saskia P. Hagenaars, Cathryn M. Lewis, Alessandro Serretti

**Affiliations:** 1 Department of Biomedical and Neuromotor Sciences, University of Bologna, Bologna, Italy; 2 Institute of Psychiatry, Psychology & Neuroscience, King’s College London, London, UK

**Keywords:** Gene expression, Predictive markers

## Abstract

Major depressive disorder (MDD) is the single largest contributor to global disability and up to 20–30% of patients do not respond to at least two antidepressants (treatment-resistant depression, TRD). This study leveraged imputed gene expression in TRD to perform a drug repurposing analysis. Among those with MDD, we defined TRD as having at least two antidepressant switches according to primary care records in UK Biobank (UKB). We performed a transcriptome-wide association study (TWAS) of TRD (*n* = 2165) vs healthy controls (*n* = 11,188) using FUSION and gene expression levels from 21 tissues. We identified compounds with opposite gene expression signatures (ConnectivityMap data) compared to our TWAS results using the Kolmogorov-Smirnov test, Spearman and Pearson correlation. As symptom patterns are routinely assessed in clinical practice and could be used to provide targeted treatments, we identified MDD subtypes associated with TRD in UKB and analysed them using the same pipeline described for TRD. Anxious MDD (*n* = 14,954) and MDD with weight gain (*n* = 4697) were associated with TRD. In the TWAS, two genes were significantly dysregulated (TMEM106B and ATP2A1 for anxious and weight gain MDD, respectively). A muscarinic receptor antagonist was identified as top candidate for repurposing in TRD; inhibition of heat shock protein 90 was the main mechanism of action identified for anxious MDD, while modulators of metabolism such as troglitazone showed promising results for MDD with weight gain. This was the first TWAS of TRD and associated MDD subtypes. Our results shed light on possible pharmacological approaches in individuals with difficult-to-treat depression.

## Introduction

Major depressive disorder (MDD) is the single largest contributor to global disability and the main contributor to suicide deaths, which number close to 800,000 per year [1]. Antidepressants are the first line treatment for moderate–severe MDD and their efficacy compared to placebo has been demonstrated by large meta-analyses;[2] however, up to 20–30% of patients develop treatment-resistant depression (TRD), i.e. their depressive symptoms do not sufficiently improve after two or more antidepressant treatments [3, 4]. TRD is associated with recurrent depression, frequent hospitalizations, negative repercussions on occupational/social functioning, decline of physical health and increased all cause-mortality [5]. A longitudinal study of TRD showed that 40% of patients had persistent depression or subsyndromal symptoms after 3 years, demonstrating the chronic course and long-term impairment associated with TRD [6].

MDD and antidepressant response have a heritable component [7, 8], the study of which can be useful to better understand the inter-individual differences in the clinical course of the disease and treatment response. Genetic studies have indeed suggested that patients with TRD have distinctive biological characteristics compared to responders [9] and the clinical heterogeneity of MDD at least partly reflects the involvement of specific genetic factors [10]. Therefore, genetic data are a precious resource to disentangle the mechanisms responsible for the heterogeneous manifestations of MDD and develop more effective treatments, i.e. treatments that target the specific biological dysfunctions found in certain groups of patients with MDD. Certain subtypes of MDD have been associated with the risk of TRD or with a chronic course and higher disability, particularly anxious, melancholic and atypical depression [11–13]. These findings confirm the hypothesis that clinical manifestations may be connected to specific biological mechanisms and to the risk of TRD. According to this hypothesis, clinical symptoms can be used not only to predict the risk of TRD, but also to guide the prescription of targeted treatments based on patterns that are easily recognizable at the first clinical assessment.

Drug repurposing guided by genetic findings is a promising approach to identify new candidate compounds for TRD and depression subtypes of interest, as selecting genetically supported targets could double the success rate in clinical development [14]. Previous studies leveraged genetic findings to identify possible drugs for repurposing in MDD (e.g. [15, 16]), while only one examined specifically TRD to the best of our knowledge and it represented a first step to the identification of drugs with genetic support of efficacy in this group of patients [17]. It used an approach based on the enrichment of drug gene targets in TRD-associated genes, therefore it could not discriminate the direction of the pharmacological effect on the disease (therapeutic or detrimental) and the results needed interpretation based on the known pathogenetic mechanisms of TRD [17]. This limitation is common also to other drug repurposing studies [18] and a possible method to address it is to impute gene expression from genome-wide trait-associated variants, then to compare imputed gene expression profiles to drug-induced gene expression profiles [16].

Therefore, in the present study we analysed UK Biobank (UKB) data with the aim to: (1) identify gene expression changes associated with TRD and depression subtypes associated with TRD risk; (2) compare the disease-associated gene expression profiles with drug-induced gene expression to select compounds that show opposite patterns of expression and could counteract the pathogenetic alterations of TRD.

## Material and methods

### Selection of depression phenotypes

We selected MDD subtypes associated with TRD and TRD itself to prioritize the identification of compounds potentially effective in cases of MDD poorly responsive to the available antidepressants and because previous studies have focused on MDD as a whole group [15, 16, 19].

TRD was defined using electronic health records (EHR) of primary care events in UKB as participants with MDD having at least two switches between different antidepressant drugs (independently from the class) satisfying the following criteria [20]:

- Each drug was prescribed for at least six consecutive weeks (noting that adequate duration for efficacy is four weeks, and our conservative threshold should reduce the risk that drug switch was due to side effects);
- The time interval between the prescription of two consecutive drugs was no longer than 14 weeks (to ensure that treatment had not been suspended).

Secondly, we tested if any MDD subtype was associated with an increased risk of TRD, considering MDD with typical and atypical neurovegetative symptoms, MDD with weight gain, anxious MDD, psychotic, seasonal, peripartum, stress-related or reactive MDD and endogenous MDD. These depression subtypes were defined using primary care EHR of diagnostic codes and/or symptoms reported as part of the mental health questionnaire (MHQ), particularly those assessed by the Composite International Diagnostic Interview Short Form (CIDI-SF) [21]; cases of bipolar, psychotic or substance use disorders were excluded and further details are available as Supplementary Methods. We decided to examine also MDD with weight gain because the subtype with atypical neurovegetative symptoms had relatively small sample size in UKB [22] and previous studies suggested that weight increase during depression drives relevant biological characteristics of this group [10]. The investigated subtypes include those previously associated with worse treatment response and worse prognosis (see “Introduction”), and the subtypes that reflect the current nosology and therefore are usually routinely assessed in the clinical practice [23]. Though reactive and endogenous depression are no longer part of the current nosology, they were present among primary care EHR as these mostly started in the 1990s, and endogenous MDD was the equivalent of melancholic depression [24]. Reactive or stress-related depression was considered because recent studies suggested it may show distinctive genetic factors compared to other MDD cases [25].

### Statistical analysis

#### Transcriptome-wide association study

We performed a genome-wide association study (GWAS) of depression subtypes found to be associated with TRD using BGENIE v1.2 and imputed genotype dosages [26]; phenotypes were residualized for six genetic ancestry principal components, assessment centre and batch effects. Sex and age were not considered as covariates as they are not risk factors for TRD according to the literature [11]. A higher average body mass index (BMI) was found in MDD cases vs healthy controls (28.34 [SD = 5.43] vs 26.43 [SD = 4.15], *p* < 5e-324), particularly in cases who reported weight gain during their worst depressive episode (30.19 [SD = 5.56]). We did not adjust the analyses for BMI, because shared genetic factors and a bi-directional relationship have been reported between MDD and overweight/obesity [27]. We included participants of European ancestry; details on quality control of genotypes are in the Supplementary Methods. Healthy controls were selected from those who completed the MHQ and/or had primary care records available and no psychiatric disorder (*n* = 54,974 after quality control). GWAS summary statistics of TRD vs healthy controls (*n* = 2,165 vs *n* = 11,188) were generated as part of a previous study [20].

SNP weights from distinct tissues and samples (of European ancestry) were used, applying the same procedure described in a recent study [28]. As in this previous transcriptome-wide association study (TWAS) of MDD, FUSION SNP-weights from postmortem brain tissue, whole blood, peripheral blood, adrenal, pituitary and thyroid glands were obtained (http://gusevlab.org/projects/fusion/#reference-functional-data). We chose to use FUSION software because it compares several models when deriving SNP-weights to account for different architecture and it has in-built functionality for conditional and colocalization analyses. We did not focus our analyses exclusively on brain tissues because: (1) a high correlation between cis-eQTLs effects in blood and brain tissues has been demonstrated, as well as a gain of power in gene discovery for brain-related phenotypes using blood cis-eQTL data with large sample sizes [29]; (2) MDD is considered a systemic disease (e.g. increased systemic inflammation, metabolic and endocrine dysfunctions) [30]; (3) Cmap mostly includes data on cancer cell lines that are not neuronal in origin [31]. The weights pertained to the following RNA reference samples: NTR (Netherlands Twin Register) and YFS (Young Finns Study), both of which provide information on blood tissue gene expression; CMC (CommonMind Consortium) and PsychENCODE Consortium, both of which assessed the dorsolateral prefrontal cortex (DLPFC); and the GTEx Consortium, which measured expression in multiple brain and peripheral tissues [32–34].

A TWAS was performed for each phenotype of interest using FUSION, setting the transcriptome-wide significance at *p* = 1.37 × 10^−6^ in line with a previous study [28]; colocalization, conditional analysis and fine mapping of significant signals were performed according to [28] and are described in the Supplementary Methods.

#### Screening of compounds for drug repurposing

As the high number of compound signatures (>130 K) available in ConnectivityMap (Cmap) and the lack of information to select a priori certain experimental conditions (e.g. cell line, dose, time of exposure), we used the Cmap Query tool (https://clue.io/query) to screen the available signatures and identify those to include in the following step (see next paragraph). To the best of our knowledge, Cmap represents the largest repository of compound-induced gene expression profiles in terms of number of available compounds and type of experimental conditions (cell lines, doses, time of exposure).

We selected the compound signatures generating gene expression profiles most dissimilar to our top TWAS results, given the hypothesis that these could counteract the alterations observed in TRD and MDD subgroups of interest [16]. Cmap catalogs transcriptional responses of human cells to chemical and genetic perturbation; >1 M replicate-collapsed signatures are available in the 2020 version of the database (Expanded CMap LINCS Resource 2020) [31]. We used the top 25, 50, 100, 150 and 250 genes dysregulated in each TWAS (with no pre-specified *p* value threshold) to identify candidate signatures generating opposite transcriptional responses, since we do not know how many genes underlie TRD or depression subtypes [16]. This method was applied in a previous study [16], although we used 25 top genes instead of 500, as we preferred to be more conservative given the relatively small size of our samples. We used the R package “biomaRt” to convert gene symbols to Entrez ID which is the nomenclature used by Cmap. When multiple features were available for the same gene (i.e. weights for multiple tissues), we selected the feature with the highest cross-validation coefficient of determination (CV *R*^2^) [35]. Cmap Query provides a measure of similarity of the provided up- or downregulated genes to those induced by perturbagens in the database, namely the connectivity score. The connectivity score represents a non-parametric similarity measure based on the weighted Kolmogorov-Smirnov (KS) enrichment statistic and it is positive for signatures that are positively related and negative for those that are inversely related [36]. To allow for comparison of connectivity scores across cell types and perturbation types, the scores are normalized by dividing a raw positive connectivity score by the mean of positive connectivity scores and a raw negative connectivity score by the mean of negative connectivity scores. A *p* value is calculated by comparing the similarity between the query and reference signature (KS test) to a null distribution of random queries and then it is adjusted for multiple testing (false discovery rate, FDR) [36].

#### Ranking of top compounds for repurposing

Selected signatures from the previous step were then examined using the approach described in a previous study that includes the R code made available by the authors [16], in order to identify those with the strongest dissimilarity to the genes dysregulated in our TWAS. In detail, the selected signatures were investigated using a combination of different methods (KS test, Spearman and Pearson correlation with all or with the top 25, 50, 100, 150 and 250 differentially expressed genes). For each compound, we calculated the average rank within each method across the examined sets of genes; then we computed the average rank across the different methods [16]. To assess the significance of the ranks, we performed permutations by shuffling the disease-expression *z*-scores and repeating the same procedure above to determine average ranks. We performed 100 permutations for each signature–phenotype pair and combined the distribution of ranks under the null across all signature–phenotype pairs; the *p* value was calculated by comparing the distribution of the permuted ranks to the observed ranks.

For these analyses we downloaded the Expanded CMap LINCS Resource 2020 level 5 data which includes replicate-collapsed *z*-score vectors representing gene expression levels [31]. We applied the following selection criteria to identify signatures of interest: (1) a negative connectivity score to our TWAS dysregulated genes; (2) FDR *p* < 0.10 (liberal threshold was used for inclusion in subsequent analyses); (3) the signature corresponded to a compound (and not the genetic loss/gain of function perturbagens included in Cmap); (4) the corresponding compound was not an antidepressant or antipsychotic medication and did not have known toxicity or relevant side effects (e.g. chemotherapy, immunosuppressant activity); (5) the signature satisfied Cmap quality control criteria [36]. If a signature satisfied all criteria but not (5), we selected an alternative signature for the same compound passing quality control and having the most similar characteristics in terms of cell line, dose of the compound and time of exposure; when more than one alternative signature having overlapping characteristics was available, we prioritized those labelled as high quality if any, otherwise we included all the available alternatives.

These analyses were performed using the R code made available by [16] in R version 4.0.3 and the R package “cmapR”.

## Results

**Fig. 1:**
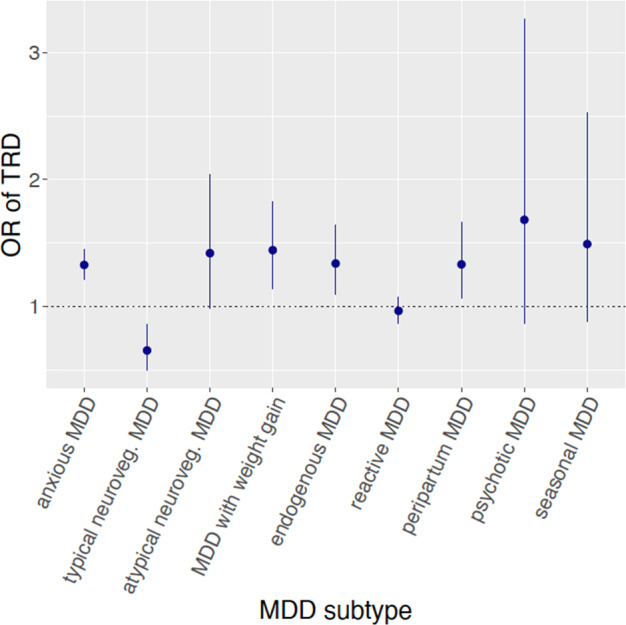
OR and 95% confidence intervals for treatment-resistant depression (TRD) in each of the examined depression subtypes. The analysed MDD subtypes were selected to reflect those with higher risk of TRD according to the previous literature and those part of the psychiatric nosology. MDD = major depressive disorder.

We identified three subtypes of MDD that were associated with increased TRD risk after Bonferroni correction, namely MDD with weight gain (*n* total = 5826 and 4697 after quality control), anxious MDD (*n* total = 18,034 and 14,954 after quality control) and endogenous MDD (*n* total 1014 and 860 after quality control) (Fig. 1). Interestingly, MDD with typical neurovegetative symptoms was associated with reduced risk of TRD. An overview of the number of cases for each subtype and their association with TRD is in Supplementary Table 1.

As the endogenous MDD subtype had a very limited sample size for further analyses, we decided to include anxious MDD, MDD with weight gain and TRD in the TWAS and subsequent analyses.

### TWAS results

**Fig. 2:**
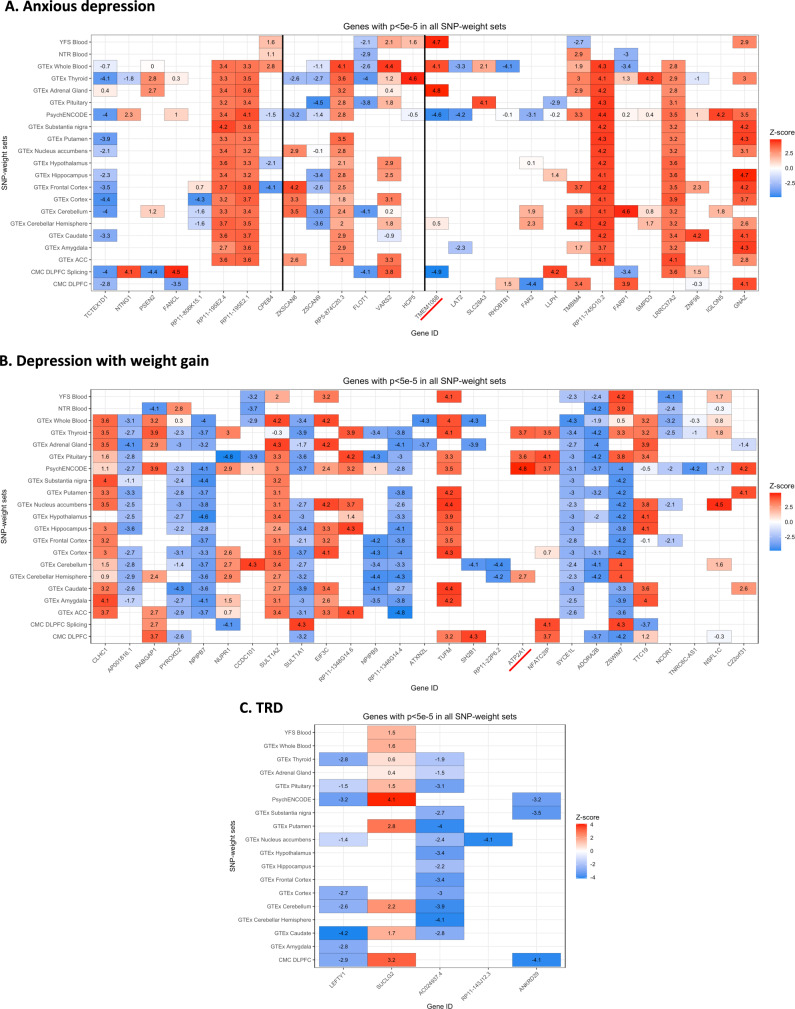
Z-scores across SNP-weight sets for genes with *p* < 5e-5. Comparisons of *z*-scores across SNP-weight sets for genes with *p* < 5e-5 for anxious depression (**A**), depression with weight gain (**B**) and treatment-resistant depression (TRD) (**C**) (described in Supplementary Table 2). White spaces correspond to genes that were not tested in the TWAS due to their not significant heritability. Blue shades indicate downregulation while red ones represent upregulation of gene expression. Black vertical lines indicate where the major histocompatibility complex region starts and ends. Transcriptome-wide significant genes are underlined in red. ACC = anterior cingulate cortex; CMC = CommonMind Consortium; DLPFC = dorsolateral prefrontal cortex; GTEx = genotype tissue expression; NTR = Netherlands Twins Register; YFS = Young Finns Study.

We identified two transcriptome-wide significant signals: TMEM106B for depression with anxiety (panel CMC DLPFC splicing, *p* = 1.23 × 10^−6^) and ATP2A1 for depression with weight gain (panel PsychENCODE, *p* = 1.34 × 10^−6^); no significant features were identified for TRD. The top features (*p* < 5e-5) for each phenotype are described in Supplementary Table 2 and *z*-scores across tissues in Fig. 2; QQ plots and Manhattan plots are in Supplementary Fig. 1.

For both TMEM106B and ATP2A1, the colocalization analysis showed that the same causal SNP was likely affecting both the risk of anxious or weight gain depression and transcription (Table 1). FOCUS suggested that both features were in the 90% credible set, though it could not identify any feature in the corresponding regions that was more likely to be causal than others (Table 1 and Supplementary Table 2). The results of the conditional analysis showed that the identified features were independent (jointly significant) (Table 1, Supplementary Table 2 and Supplementary Fig. 2).

**Table 1:** Transcriptome-wide significant genes in at least one tissue; we reported a comparison with the latest TWAS of major depressive disorder (MDD) (Dall’Aglio et al., 2021 [28]) and with previous GWASs of neuropsychiatric and cardio-metabolic traits (according to GWAS Catalog: https://www.ebi.ac.uk/, accessed on 4th May 2021; GWAS Catalog annotations are based on the last Ensembl release, genes in which a variant maps are reported or the closest upstream or downstream gene within 50 kb).

Phenotype	Gene	Panel	*z-*score	*p* value	PP3	PP4	In FOCUS credibility set	Significant in previous MDD TWAS	Traits with significant associations in previous GWASs
Anxious MDD	TMEM106B	CMC DLPFC splicing	−4.85	1.23e-6^a^	0.022	0.972	Yes	No	Neuroticism, feeling tense, mood swings, depressive symptoms, depression, frontotemporal dementia, aging, help-seeking from a GP, years of education, well-being spectrum, anxiety disorders, cardio-metabolic traits
		GTEx adrenal gland	4.79	1.65e-6	0.013	0.978	Yes	Yes	
		YFS blood	4.67	3.02e-6	0.011	0.986	Yes	Yes	
		PsychENCODE	−4.63	3.58e-6	0.062	0.934	Yes	Yes	
		GTEx whole blood	4.05	5.11e-5	–	–	No	Yes	
		GTEx cerebellar hemisphere	0.47	0.64	–	–	No	No	
MDD with weight gain	ATP2A1	PsychENCODE	4.83	1.34e-6^a^	0.084	0.875	Yes	No	Cannabis use, risk taking behaviour, bipolar disorder, schizophrenia, intelligence, years of education, cardio-metabolic traits
		GTEx thyroid	3.69	2.23e-4	–	–	Yes	No	
		GTEx pituitary	3.57	3.64e-4	–	–	Yes	No	
		GTEx cerebellar hemisphere	2.68	7.45e-3	–	–	No	No	

Posterior probability PP3 = probability that two causal SNPs in linkage disequilibrium affect transcription and the phenotype separately; posterior probability PP4 = probability that the same causal SNP affects both transcription and the phenotype. *CMC* CommonMind Consortium, *DLPFC* dorsolateral prefrontal cortex, *GTEx* genotype tissue expression, *YFS* Young Finns Study, *GP* general practitioner.

^a^Transcriptome-wide significant results.

The top 250 genes for each phenotype available in Cmap are in Supplementary Table 3.

### Candidate compounds for repurposing

The total number of examined signatures of compound perturbagens was 136,460, which corresponded to 29,679 unique compounds. A total of 76, 41 and 21 compounds showed a negative connectivity score and FDR *p* < 0.10 for TRD, depression with anxiety and depression with weight gain, respectively (Supplementary Table 4). They included four approved psychotropic medications (one antidepressant, two antipsychotics and one antiepileptic).

For TRD, the most common mechanisms of action of the identified compounds were modulation of cell survival–proliferation–differentiation (13%) and monoaminergic neurotransmission (7%). For anxious depression, modulation of cell survival–proliferation–differentiation was again the most common mechanism of action (15%), but interestingly inhibition of heat shock proteins (HSP) was the second one (10%). Candidate compounds for depression with weight gain included mostly modulators of metabolism (14%), such as peroxisome proliferator-activated receptor (PPAR) agonism. A relatively high proportion of the identified compounds had no reported mechanisms of action in the Cmap database (53%, 39% and 67% for TRD, depression with anxiety and depression with weight gain, respectively).

### Ranking of candidate compounds for repurposing

For TRD, anxious and weight gain MDD, respectively, a total of 58, 35 and 18 compounds had at least one available expression signature passing quality control (Supplementary Table 4). For TRD we identified three compounds with permuted *p* < 0.05, which included zamifenacin (a muscarinic M3 and M5 receptor antagonist) and two molecules with unknown mechanism of action.

Four compounds showed permuted *p* < 0.05 for anxious depression: two were HSP90 inhibitors, one was a miR122 inhibitor and one was a modulator of cell cytoskeleton. Finally, for MDD with weight gain, only one signature corresponding to a molecule with unknown mechanism of action had significant permuted (BRD-K60636255). The top results for each group are summarized in Table 2.

**Table 2:** Compounds showing an oppositive transcriptomic profile to the expression profiles imputed in TRD, anxious MDD and MDD with weight gain vs healthy controls.

Phenotype	Compound	Signature ID	*p* value	MOA	Previous findings
TRD	Zamifenacin	ERG021_PC3_24H.BRD.K80451230.3.33	0.002	Acetylcholine M3 and M5 receptor antagonist	Scopolamine has AD effects in TRD [46] but antagonisms on M2 seems to be responsible for the AD effects, though more data are needed to clarify the role of other muscarinic receptor subtypes [48]
	BRD-K92033419	CPC010_HT29_6H.BRD.K92033419.001.06.7.10	0.004	Unknown	/
	BRD-K95664364	DOS005_VCAP_6H.BRD.K95664364.001.01.2.4.79	0.039	Unknown	/
	Dantrolene	CPC017_ASC_24H.BRD.K81272440.236.06.9.10	0.061	Calcium channel blocker	It shows also inhibition of monoamine oxidase B and acetylcholinesterase, it has neuroprotective effects [57]
	Zardaverine	CPC016_HEPG2_6H.BRD.K37561857.001.06.4.10	0.065	PDE4 and PDE3 inhibitor	PDE4 and PDE3 inhibition has AD-like effects [58]
	BRD-K09661167	CPC012_ASC_24H.BRD.K09661167.001.06.1.10	0.065	RAR-related orphan receptor gamma inhibitor, Bcl2-A1 Inhibitor, NOD1 inhibitor	/
	BRD-K39284479	DOSBIO001_PC3_24H.BRD.K39284479.10.0519	0.070	Unknown	/
	Zebularine	CPC006_HEPG2_6H.BRD.A01145011.001.01.4.11.1	0.073	DNA methylation inhibitor	It reverses the behavioural deficits induced by chronic stress [59]
Anxious MDD	Tanespimycin	MUC.CP007_P1A82_24H.G17	0.009	HSP90 inhibitor	HSP90 inhibition increases lifespan and health in animal models [50]. FKBP51, acting via HSP90, is a pathogenetic mechanism in MDD [51]
	SNX-2112	REP.B022_HA1E_24H.I01	0.013	HSP90 inhibitor	See tanespimycin
	Cytochalasin-d	CPC013_HCC515_6H.BRD.K25504083.001.03.1.10	0.016	Tubulin inhibitor	It acts as inhibitor of the G-actin–cofilin interaction [60] that has a detrimental role in neurodegenerative disorders as it promotes neuronal cell death [55]. Cytochalasin-d increases the clearance of neuron-released α-synuclein by microglia [61]
	BRD-K85392418	MUC.CP007_P1A82_24H.G17	0.026	miR122 inhibitor	miR122 activates HSP70 and NF-κB pathway; NF-κB signalling activated by stress has a role in the pathogenesis of depressive behaviours [54]
	Azithromycin	REP.B024_HA1E_24H.B22	0.062	Bacterial 50 S ribosomal subunit inhibitor	β-lactam antibiotics were found to promote the expression of the glutamate transporter GLT1 and have a neuroprotective role [62]
MDD with weight gain	BRD-K60636255	DOSBIO001_NPC_24H.BRD.K60636255.10.0166	0.025	Unknown	Unknown

Ranking was obtained by averaging the results of a KS test, Spearman correlation with all or with the most differentially expressed genes and Pearson correlation with all or with the most differentially expressed genes; 100 permutations were performed to assess the significance of the ranks (permuted *p* values < 0.10 are reported). *MOA* mechanisms of action, *AD* antidepressant.

## Discussion

This study leveraged imputed gene expression profiles of TRD and associated depression subtypes to identify compounds showing opposite transcriptomic changes, which may have therapeutic effects in these groups as they show poor response to standard treatments.

Our TWAS findings for anxious MDD found that TMEM106B expression was downregulated in brain tissues, in line with the results of a recent TWAS of MDD [28]; in total, ten of our top findings for anxious MDD (*p* < 5e-5) were significantly dysregulated in the same study (see Supplementary Table 2 for a comparison of our results with the previous MDD TWAS [28]). In previous GWASs, SNPs in TMEM106B have been associated with MDD (e.g. the intronic SNP rs10950398 [7]), anxiety disorders (2 kb upstream variant rs3807866 [37]) and other traits (Table 1). The intronic rs5011432 SNP in TMEM106B has been associated with MDD in a previous GWAS [38], and it was the strongest eQTL for the TMEM106B TWAS association with anxious MDD (YFS BLOOD).

Interestingly, we found that ATP2A1 showed significant transcriptomic-wide association with MDD with weight gain, and this gene was implicated in GWASs of anthropometric traits such as BMI, type 2 diabetes, cognitive abilities, cannabis use, schizophrenia and bipolar disorder, therefore it probably represents a pleiotropic genetic factor regulating both the risk of metabolic and psychiatric disorders [39–43] (Table 1). This study was the first to determine the significance of ATP2A1 on a psychiatric trait using a TWAS to the best of our knowledge. The strongest eQTL for ATP2A1 was associated with cannabis use in a previous GWAS [41] (rs10499, GTEx pituitary).

Our screening of molecules for drug repurposing in TRD identified 76 compounds, one of which was shared with anxious MDD, two were antipsychotics, and six were identified also in a previous drug repurposing study for TRD [17], though the latter applied a different methodology and compared TRD vs antidepressant responders. However, the distribution of the mechanisms of action between the two studies was similar, with a prevalence of compounds modulating cell survival, proliferation, differentiation, monoaminergic neurotransmission and inflammation. One of the identified drugs, aprepitant, is a tachykinin antagonist and it failed phase III clinical trials for depression, but it has been suggested that insufficient doses were employed based on PET occupancy data [44]. Other identified compounds have known antidepressant-like or neuroprotective effects, such as vincamine, bergenin, zebularine, zardaverine, dantrolene, clenbuterol and stiripentol (Supplementary Table 4). The ranking of compounds combining the connectivity score, Spearman and Pearson correlation, identified three molecules with significant permuted *p* value, zamifenacin (a muscarinic M3 and M5 receptor antagonist) and two compounds with still unknown activity. Some antidepressants and atypical antipsychotics show M3 receptor antagonism, and scopolamine, a non-selective antimuscarinic drug, shows rapid antidepressant effects in TRD [45, 46]. However, the positive effects on mood are mostly attributed to antagonism on the M2 receptor subtype [47], but the knowledge about the role of M3 and M5 receptor subtypes is still limited [48]. Dantrolene (a calcium channel blocker) and zebularine (a modulator of DNA methylation) were very close to the significance threshold and have previous evidence of involvement in pathways relevant to MDD (Table 2).

For anxious depression, one of the 41 compounds identified in the screening phase was an approved antidepressant (agomelatine) and two were identified in the previously cited TRD repurposing study [17]. The modulation of cell survival/proliferation was again the most common known effect of the identified molecules, but another relatively common mechanism was the inhibition of HSP90, and two compounds with this effect were among the top ranking drugs in the following step of the analysis (SNX-2112 and tanespimycin). HSP expression is associated with stress response and HSP effects are complex: they can be pro-cell survival or pro-death depending on the molecules they interact with, on the tissue, on the cell type [49]. Interestingly, HSP90 inhibition (including by tanespimycin) increases lifespan and health in animal models [50]. The available studies suggest that although increased HSP levels may be beneficial for acute conditions, such increases can be detrimental for chronic conditions, as exemplified by acute and chronic heart conditions [51]. In regard to MDD, the co-chaperone FKBP51, acting via HSP90, is an established pathogenetic mechanism, through the induction of glucocorticoid resistance and a poor stress coping phenotype [51]. BRD-K85392418 was another of the identified compounds for anxious MDD and it was reported to act as miR122 inhibitor. This micro-RNA was found to be altered in MDD [52] and it increases the activity of HSP70 and the NF-κB pathway, which have protective effects during acute stress conditions [53], and also in this case their activity has likely detrimental effects in the long term as they upregulate proinflammatory cytokine expression and cause impaired neurogenesis [54]. The last significant compound for repurposing in anxious MDD was cytochalasin-d, which acts as inhibitor of the G-actin–cofilin interaction, a process that has been implicated in neurodegenerative disorders as it promotes neuronal cell death [55].

Among the 20 molecules identified in the screening step for MDD with weight gain, one was also significant in our previous study of drug repurposing in TRD [17]. This was troglitazone, a proliferator-activated receptor (PPAR) agonist which was among the most promising results since PPAR agonists showed antidepressant effects in four open-label studies and in three out of four RCTs in patients with major depression [56]. However, troglitazone did not show significant permuted *p* value in the drug ranking using the connectivity score, Spearman and Pearson correlation, and this analysis identified only one molecule with unknown mechanism of action (Table 2).

Our results should be interpreted in the light of the limitations of this study. The sample sizes included in the TWAS were relatively limited, as they were constrained to the number of cases available for each MDD subtype and TRD, and to the number of participants of European ancestry. The weights used in the TWAS were calculated based on existing eQTL datasets, which were often obtained in small samples, particularly for brain tissues in GTEx, though PsychENCODE is larger. On the other hand, the use of imputed gene expression from GWAS is much more feasible on a large scale than the direct measurement of gene expression levels. The tested tissues were selected based on the previous literature, however, the inclusion of 21 distinct SNP-weight sets from different tissues might capture noncausal genes [28]. The restriction of the analysis to brain tissues could be an alternative option, particularly on large datasets; however, Cmap expression signatures were mostly obtained in non-neural cell lines. Our TWAS approach solely assessed the cis-genetic component of gene expression, while it could not capture trans-eQTL effects. Finally, the approach used for drug repurposing was based on the dissimilarity of gene expression profiles in the TWAS and Cmap compound-induced expression profiles; however, the latter were determined in vitro and under heterogenous experimental conditions (different cell lines, different drug dose and time of exposure), and we did not know which ones were more similar to in vivo gene expression changes.

In conclusion, our study identified two genes showing transcriptomic alterations in depression subtypes associated with TRD: TMEM106B in anxious MDD and ATP2A1 in MDD with weight gain; both genes had previous evidence of involvement in psychiatric traits and also metabolic traits in the case of ATP2A1. Our drug repurposing analyses suggested that the inhibition of HSP90 and the modulation of cell cytoskeleton may represent alternative strategies for the treatment of anxious MDD, while drugs modulating metabolism may be beneficial in MDD with weight gain. For the treatment of TRD, potentially useful pharmacological mechanisms included the antagonism of muscarinic receptors, the modulation of DNA methylation and calcium channels.

## Supplementary information

Supplementary Methods-Table S1 - Figures.

Supplementary Table 2.

Supplementary Table 3.

Supplementary Table 4.
