# Supplementary material for: Transcriptome-wide association study of treatment-resistant depression and depression subtypes for drug repurposing
Source: Neuropsychopharmacology. 2021 Jun 22;46(10):1821–9. doi: 10.1038/s41386-021-01059-6 (PMC8357803; doi:10.1038/s41386-021-01059-6)

Transcriptome-wide association study of treatment-resistant depression and depression subtypes for drug repurposing

Table of contents

Supplementary Methods………………………………………………………………………………….. p. 1
Supplementary Table 1…………………………………………………………………………………… p. 4
Supplementary Figure 1………………………………………………………………………………… p. 5
Supplementary Figure 2………………………………………………………………………………… p. 8

Supplementary Methods

1. Definition of depression subtypes

For all major depressive disorder (MDD) subtypes we excluded individuals who reported a history of psychotic, bipolar and substance use disorders according to the mental health questionnaire or primary care records.

Depression with typical neurovegetative symptoms: individuals with a history of lifetime MDD according to the Composite International Diagnostic Interview Short Form (CIDI-SF) [1] who reported appetite/weight decrease and insomnia during the depressive episode according to the CIDI-SF [2].

Depression with atypical neurovegetative symptoms: individuals with a history of lifetime MDD according to the CIDI-SF [1] who reported appetite/weight increase and hypersomnia during the depressive episode according to the CIDI-SF [2].

Depression with weight gain: individuals with a history of lifetime MDD according to the CIDI-SF [1] who reported weight increase during the depressive episode according to the CIDI-SF.

Depression with anxiety: individuals satisfying one of the following:

1) a history of lifetime MDD according to the CIDI-SF [1] and at least two anxiety traits according to the short version of the Eysenck Personality Inventory Neuroticism scale (tense/'highly strung'; worrier/anxious feelings; nervous feelings) [3] combined with trouble falling asleep during the reported depressive episode according to the CIDI-SF.
2) At least one diagnostic code for anxious depression according to primary care records (Read v3 codes E2003, Eu412 and X00Sb).

Peripartum depression: individuals satisfying one of the following:

1) At least one diagnostic code for post-partum depression according to primary care records (Read v3 codes XaY2C, XE1aY and E204. and Eu32B).

2) Having reported depression possibly related to childbirth (data field 20445) and a history of lifetime MDD according to the CIDI-SF [1].

Psychotic, seasonal and endogenous depression: At least one diagnostic code for the corresponding depression subtype according to primary care records (Read v3 codes for psychotic depression: XE1ZZ, XSGon, XE1Ze, Eu323, Eu333; Read v3 code for seasonal depression: X761L; Read v3 codes for endogenous depression: X00SR, X00SS and XM1GC).

Stress-related or reactive depression: participants satisfying one of the following:

1) a history of lifetime MDD according to the CIDI-SF [1] and having reported that depression started within two months after a stressful or traumatic event (data field 20447).
2) At least one diagnostic code for reactive depression according to primary care records (Read v3 code XE1YC).

Treatment-resistant depression

Among participants with MDD according to primary care records, we defined treatment-resistant depression (TRD) as having at least two switches between different antidepressant drugs (independently from the class) satisfying the following criteria [4]:

- Each drug was prescribed for at least six consecutive weeks to ensure adequate duration for efficacy;
- The time interval between the prescription of two consecutive drugs was no longer than 14 weeks (to ensure that treatment had not been suspended).

2. Genotyping, quality control and imputation

Genome-wide genotyping on all UK Biobank participants was performed using two highly overlapping arrays covering ~800,000 markers. Autosomal genotype data underwent centralised quality control to adjust for possible array effects, batch effects, plate effects, and departures from Hardy-Weinberg equilibrium (HWE) [5]. SNPs were further excluded based on missingness (> 0.02) and on Hardy Weinberg equilibrium ($p < 10e-8$). Individuals were removed for high levels of missingness (> 0.05) or abnormal heterozygosity (as defined during centralised quality control), relatedness of up to third-degree kinship (KING $r < 0.044$ [6]) or phenotypic and genotypic gender discordance. Population structure within the UK Biobank cohort was assessed using principal component analysis, with European ancestry defined by 4-means clustering on the first two genetic principal components [7].

A two-stage imputation was performed using the Haplotype Reference Consortium (HRC) and UK10K reference panels [5] [8] [9]. Poor imputed variants were excluded (INFO ≤ 0.4) [8].

3. Transcriptome-wide association study: colocalization, conditional analysis and fine mapping

These analyses were performed following the same procedure described a recent study [10].

We used the coloc R package to assess colocalization for genes meeting transcriptome-wide significance and within a 1.5-Mb window. This Bayesian approach differentiates between associations driven by horizontal pleiotropy (one causal SNP affecting both transcription and the

phenotype; posterior probability PP4) and linkage (two causal SNPs in linkage disequilibrium affecting transcription and the phenotype separately; posterior probability PP3).
We used FUSION to perform a conditional analysis in order to determine whether multiple significant features within a given locus represented independent associations or a single association owing to correlated predicted expression between features. Independent associations are termed jointly significant, while features that are not significant when accounting for the predicted expression of other features in the region are termed marginally significant.
We used FOCUS to identify which features are likely to be causal within regions of association [11]. FOCUS estimates the posterior inclusion probability (PIP) of each feature being causal within a region of association, using the sum of posterior probabilities to define the default 90% credible set, a set of features likely to contain the causal feature. The method includes a null model where the causal feature is not present. The PIP of individual features is also of interest, with values > 0.5 indicating that a feature is more likely to be causal than any other feature in the region. The FOCUS fine mapping function was applied across all SNP weight panels simultaneously without the tissue prioritization option.

References

1. Davis KAS, Coleman JRI, Adams M, Allen N, Breen G, Cullen B, et al. Mental health in UK Biobank - development, implementation and results from an online questionnaire completed by 157 366 participants: a reanalysis. BJPsych Open. 2020;6:e18.
2. Badini I, Coleman JRI, Hagenaars SP, Hotopf M, Breen G, Lewis CM, et al. Depression with atypical neurovegetative symptoms shares genetic predisposition with immuno-metabolic traits and alcohol consumption. Psychol Med. 2020:1–11.
3. Gale CR, Čukić I, Batty GD, McIntosh AM, Weiss A, Deary IJ. When Is Higher Neuroticism Protective Against Death? Findings From UK Biobank. Psychol Sci. 2017;28:1345–1357.
4. Fabbri C, Hagenaars SP, John C, Williams AT, Shrine N, Moles L, et al. Genetic and clinical characteristics of treatment-resistant depression using primary care records in two UK cohorts. Mol Psychiatry. 2021. 22 March 2021. https://doi.org/10.1038/s41380-021-01062-9.
5. Bycroft C, Freeman C, Petkova D, Band G, Elliott LT, Sharp K, et al. The UK Biobank resource with deep phenotyping and genomic data. Nature. 2018;562:203–209.
6. Manichaikul A, Mychaleckyj JC, Rich SS, Daly K, Sale M, Chen W-M. Robust relationship inference in genome-wide association studies. Bioinforma Oxf Engl. 2010;26:2867–2873.
7. Warren HR, Evangelou E, Cabrera CP, Gao H, Ren M, Mifsud B, et al. Genome-wide association analysis identifies novel blood pressure loci and offers biological insights into cardiovascular risk. Nat Genet. 2017;49:403–415.
8. McCarthy S, Das S, Kretzschmar W, Delaneau O, Wood AR, Teumer A, et al. A reference panel of 64,976 haplotypes for genotype imputation. Nat Genet. 2016;48:1279–1283.
9. UK10K Consortium, Walter K, Min JL, Huang J, Crooks L, Memari Y, et al. The UK10K project identifies rare variants in health and disease. Nature. 2015;526:82–90.
10. Dall'Aglio L, Lewis CM, Pain O. Delineating the Genetic Component of Gene Expression in Major Depression. Biol Psychiatry. 2021;89:627–636.
11. Mancuso N, Freund MK, Johnson R, Shi H, Kichaev G, Gusev A, et al. Probabilistic fine-mapping of transcriptome-wide association studies. Nat Genet. 2019;51:675–682.

Supplementary Table 1: number of cases for each depression subtype and association with TRD. See also Figure 1 for a representation of association with TRD risk. *significant results after Bonferroni correction.

**Depression subtype**	**Yes/no total**	**Yes/no with available TRD information**	**Association with TRD (p Bonf. corrected)**
Typical neurovegetative symptoms	10,808/20,629	837/1,772<br>9% and 13.1% had TRD, respectively	OR=0.65 (0.50-0.86)<br>p=2.13e-2*
Atypical neurovegetative symptoms	1,740/29,697	253/2,356<br>15.4% and 11.4% had TRD, respectively	OR=1.42 (0.99-2.04)<br>p=0.53
With weight gain	5,826/25,610	640/3,461<br>16.3% and 11.8% had TRD, respectively	OR=1.44 (1.14- 1.82)<br>p=1.88e-2*
Anxious depression	18,034/34,711	6,342/12,036<br>15.4% and 12.1% had TRD, respectively	OR=1.33 (1.22-1.45)<br>p=2.01e-9*
Peripartum depression	3,230/60,311	568/17,810<br>16.7% and 13.1% had TRD, respectively	OR=1.33 (1.06-1.67)<br>p=0.11
Psychotic depression	72/36,808	54/18,324<br>20.4% and 13.2% had TRD, respectively	OR=1.68 (0.87-3.27)<br>p=1
Seasonal depression	124/36,756	92/18,286<br>18.5% and 13.2% had TRD, respectively	OR=1.49 (0.88-2.53)<br>p=1
Endogenous depression (melancholic)	1,014/35,866	704/17,674<br>16,8% and 13.1% had TRD, respectively	OR=1.34 (1.09-1.64)<br>p=4.29e-2*
Stress-related or reactive depression	27,315/36,151	3,929/14,449<br>12.9% and 13.3% had TRD, respectively	OR=0.97 (0.87-1.07)<br>p=1

Supplementary Figure 1: QQ plots and Manhattan plots for anxious depression (**A**), depression with weight gain (**B**) and treatment-resistant depression (**C**).

A

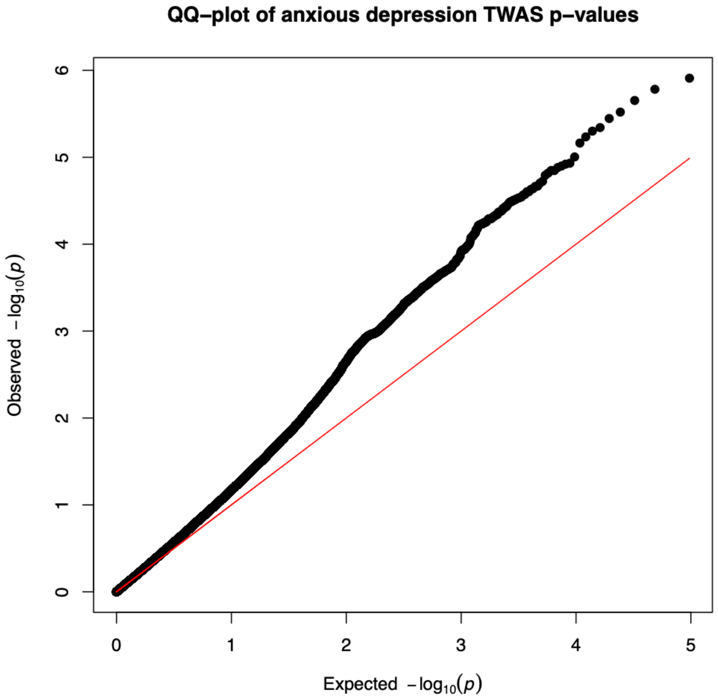

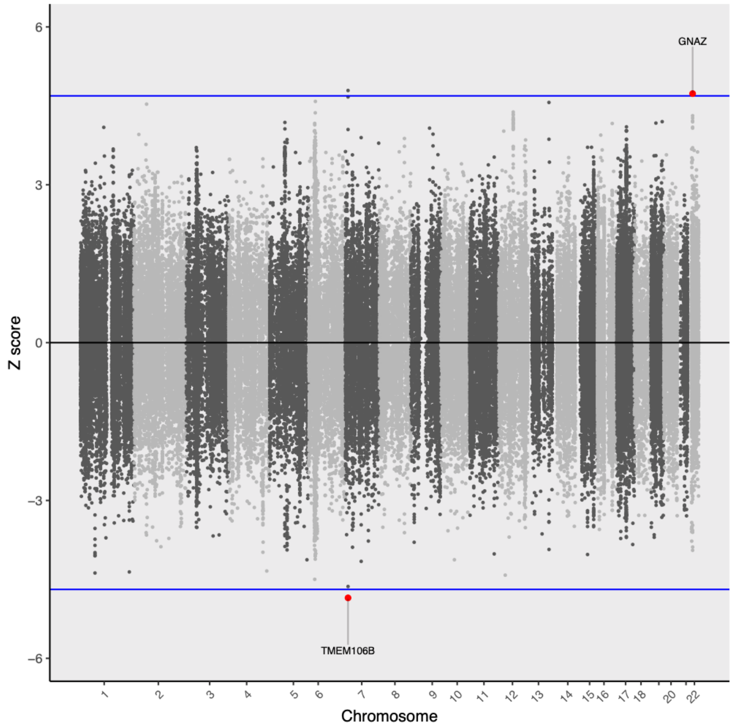

B

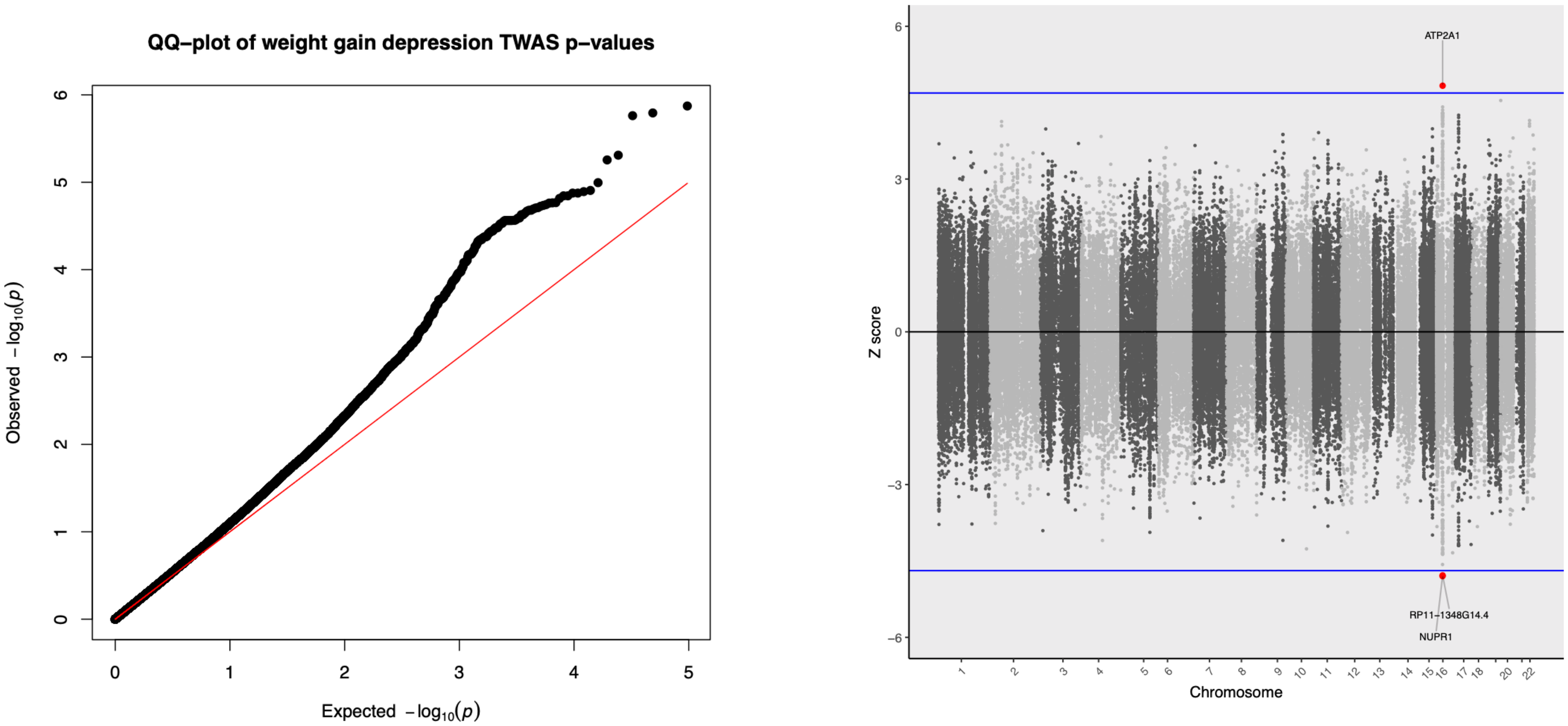

QQ–plot of weight gain depression TWAS p–values
Observed −log10(p)
Expected −log10(p)
0
1
2
3
4
5
6
ATP2A1
RP11−1348G14.4
NUPR1
Z score
Chromosome

c

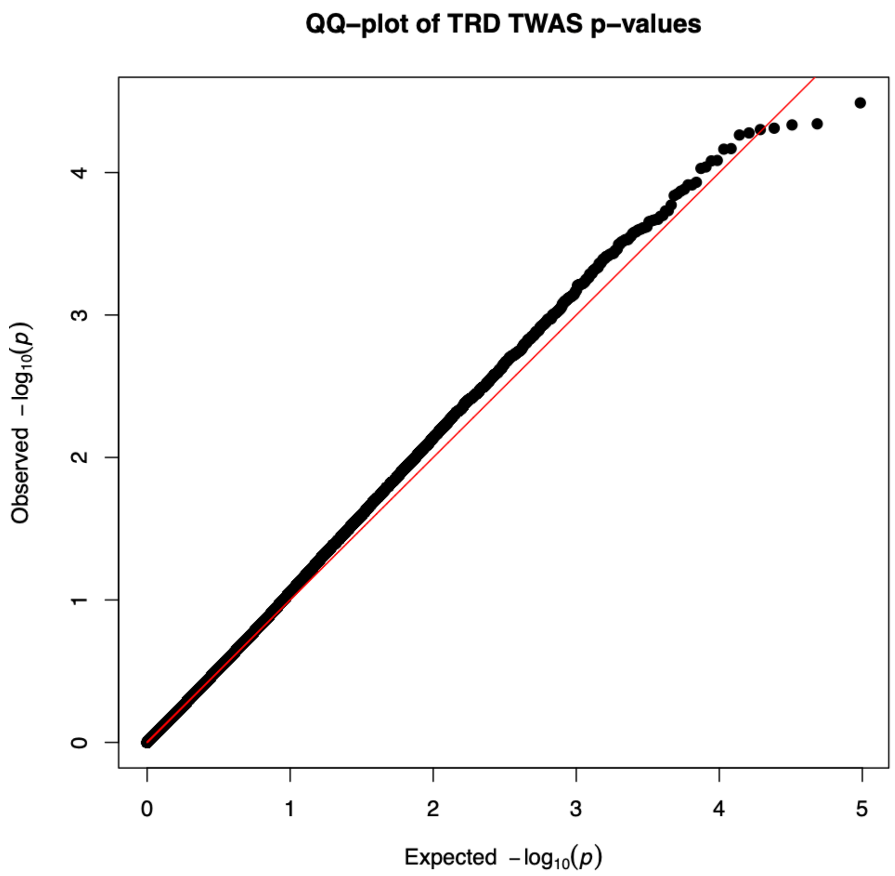
QQ–plot of TRD TWAS p–values
Observed $-\log_{10}(p)$
Expected $-\log_{10}(p)$
0
1
2
3
4
5

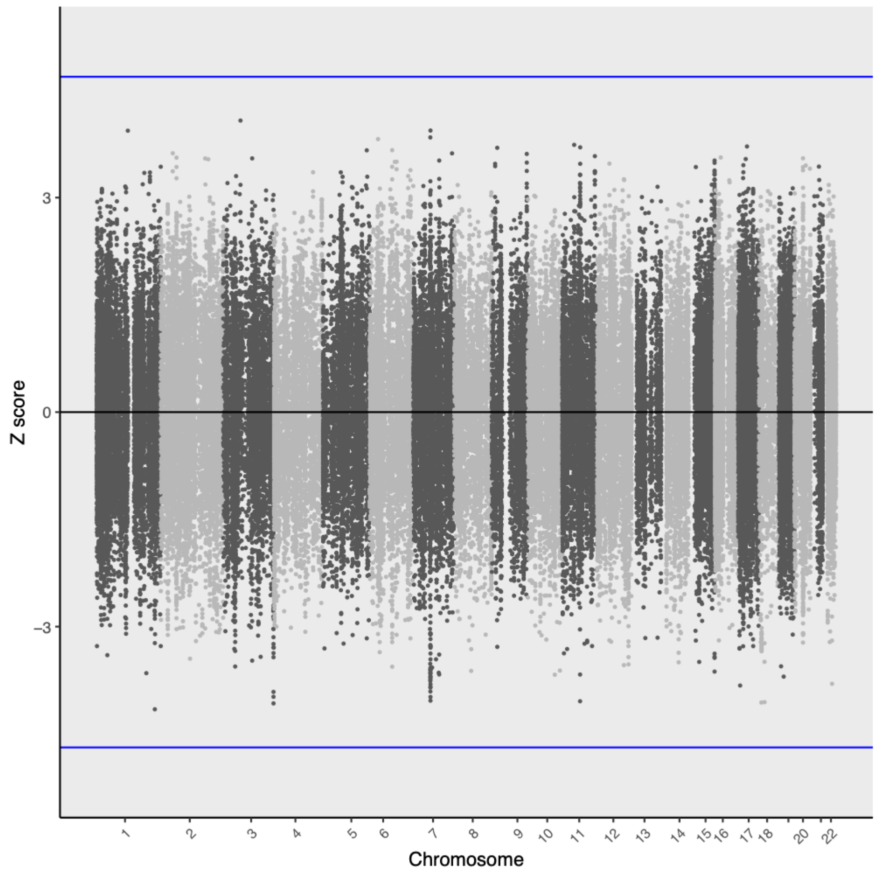
Z score
Chromosome
3
0
-3
1
2
3
4
5
6
7
8
9
10
11
12
13
14
15
16
17
18
20
22

Supplementary Figure 2: results of the conditional analysis for the TWAS significant genes for anxious MDD (**A**) and MDD with weight gain (**B**). The top panel shows all of the genes in the locus. The marginally TWAS associated genes are highlighted in blue, and those that are jointly significant are highlighted in green.

A

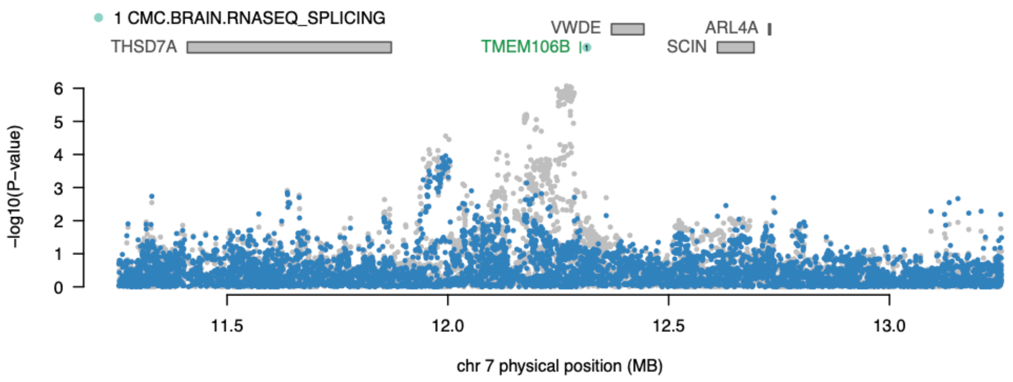

B

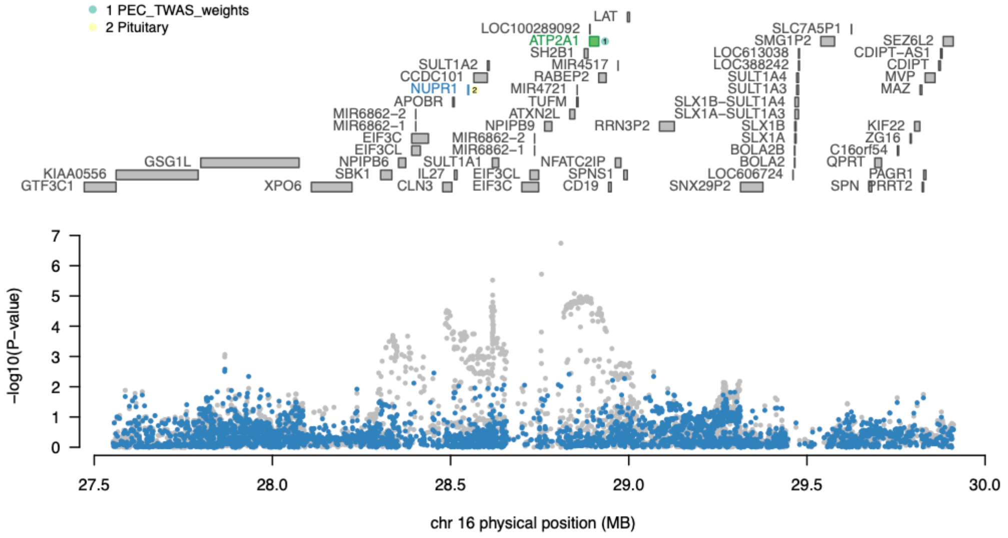

Supplement: Supplementary file 1 — Supplementary Methods-Table S1 - Figures. [file 41386_2021_1059_MOESM1_ESM.pdf]
